# Effects of Immobilized Antimicrobial Peptides on Growth Performance, Serum Biochemical Index, Inflammatory Factors, Intestinal Morphology, and Microbial Community in Weaning Pigs

**DOI:** 10.3389/fimmu.2022.872990

**Published:** 2022-03-29

**Authors:** Nian Liu, Xiaokang Ma, Xianren Jiang

**Affiliations:** ^1^ College of Animal Science and Technology, Hunan Agricultural University, Changsha, China; ^2^ Key Laboratory of Feed Biotechnology of Ministry of Agriculture and Rural Affairs, Institute of Feed Research, Chinese Academy of Agricultural Sciences, Beijing, China

**Keywords:** immobilized antimicrobial peptides, growth performance, serum biochemical index, inflammatory factors, intestinal morphology, microbial community, weaning piglets

## Abstract

This experiment was conducted to investigate the effects of immobilized antimicrobial peptides on growth performance, serum biochemical index, inflammatory factors, intestinal morphology, and microbial community of weaning piglets. A total of 21 weaning piglets [Duroc × (Landrace × Yorkshire)] with initial body weight (7.64 ± 0.65 kg) were randomly allocated to one of three treatments with seven replicates (one pig per replicate) per treatment according to sex and weight in randomized complete block design. Pigs in the three treatments were fed corn–soybean meal-based diet (CON), corn–soybean meal based diet + flavomycin (25 mg/kg) + quinone (50 mg/kg) (AB), and corn–soybean meal based diet + 1,000 mg/kg immobilized antimicrobial peptides (IAMPs), respectively. The experiment lasted for 28 days, including early stage (0–14 days) and late stage (15–28 days). The results showed the following: (1) compared with the CON group, the average daily gain in the whole experimental time (*p* < 0.05) was significantly increased, and the diarrhea rate of weaning piglets was decreased (*p* < 0.01) in the IAMPs group; (2) compared with the CON group, the concentrations of serum IgM and superoxide dismutase (SOD) in the IAMPs group were significantly higher than the CON and AB groups (*p* < 0.01); (3) compared with CON group, the concentrations of serum interleukin (IL)-10 and transforming growth factor (TGF-β) were significantly increased (*p* < 0.05), and the concentration of IL-12 was significantly decreased (*p* < 0.05) in the IAMPs group; (4) compared with CON group, the concentrations of serum endotoxin and D-lactate of piglets were significantly reduced (*p* < 0.05), and the relative expression of ZO-1 and occludin in the jejunum of piglets were significantly increased (*p* < 0.05) in the IAMPs group; (5) compared with the CON group, the villus height of the duodenum and jejunum of weaning piglets in IAMPs and AB groups was significantly increased (*p* < 0.05); and (6) compared with CON group, the relative abundance of *Escherichia–Shigella* in the colon and cecal digesta was decreased. In summary, the addition of 1,000 mg/kg immobilized antimicrobial peptides in the diet effectively relieved weaning stress by showing improved growth performance, antioxidant and immune capacity, intestinal morphology, and microorganisms.

## Introduction

The health status of weaning piglets plays a key role in improving production performance and economic benefits in the later fattening stage. However, the digestive tract and intestinal barrier of weaning piglets is not fully developed. Weaning piglets changed fodder from digestible milk to solid feed, which is difficult for piglets to digest; moreover, plant-based feeds contain antinutritional factors, and the secretion of digestive enzymes is insufficient. The microorganisms composition in the environment also change; consequently, weaning piglets cannot adapt to these changes in a short period of time, and the “weaning stress” is easily happens (1, 2). It is well known that weaning stress can cause diarrhea, growth retardation, intestinal flora disorder, and even the death of piglets, which bring a huge impact on pig breeding industry (3). Antibiotics are widely used to prevent and treat animal diseases, whereas the abuse of antibiotics leads to drug resistance and antibiotic residues in livestock and poultry and causes serious harm to human health (4). Since July 1, 2020, the era of complete prohibition in antibiotics has arrived which made the development of new feed additives imminent.

Antimicrobial peptides (AMPs) are small proteins with a wide range of sources and are one of the most common additives to substitute antibiotics at the current research (5). As an important part of the innate immune system, AMPs play an important role in the immune functions of the body. AMPs have both antibacterial and antiviral activities and will not cause drug resistance in livestock and poultry, which is normally seen in antibiotics (6, 7). Wang et al. (8) found that lactoferrin antimicrobial peptide could increase the relative abundance of beneficial bacteria such as *Lactobacillus* and *Bifidobacterium* and reduce the abundance of harmful bacteria such as *Salmonella* in the intestinal tract of weaning piglets. At the same time, some studies reported that porcine AMPs can enhance the proliferation of splenic lymphocytes and increase the levels of immunoglobulin and anti-inflammatory factors in serum of pigs (9, 10); Yu et al. (11) proved that microcin J25 supplementation in weaning piglets diet can effectively improve growth performance, relieve diarrhea and systemic inflammation, improve intestinal health, and regulate intestinal microflora structure. However, AMPs could be vulnerable to heat and other stringent environmental conditions. Most AMPs are highly susceptible to digestive enzymes. Carmona et al. (12) reported that L-pin2 is rapidly divided in two proteolytic fragments in the presence of trypsin. Naimi et al. (13) proved through experiments that MccJ25 will be degraded by pancreatic elastase and lost its antibacterial activity as soon as it enters the duodenum. This limits the effect of administrating AMPs in the feed. AMPs may lose their antimicrobial activity during storage, feed processing, and digestion in the small intestine of the animals. Immobilization is a technique to covalently bond target molecule on a solid carrier, protecting the target molecule against enzymatic degradation, denaturants, and hash environment. Immobilized antimicrobial peptides (IAMPs) have remarkably higher longer-term stability, and higher resistance to heat and proteolytic attack of enzymes comparing to soluble AMPs (14). Appendini and Hotchkiss (15) reported that IAMP could maintain its antimicrobial activity after treatment with autoclaving at 121°C for 15 min or dry heating at 200°C for 0.5 h. Haynie et al. (16) reported that IAMPs degrade two or three decades slower than soluble AMPs under protease treatment. In comparison, IAMPs have greater development potential than traditional AMPs and can play a more stable role in livestock and poultry health.

In recent years, with the increase in people’s attention to the abuse of antibiotics and food safety, AMPs as a biological peptide have been developed to a certain extent, but there are rarely studies on the application of IAMPs in feed industry. In this study, the effects of IAMPs on growth performance, immune status, antioxidant performance, and intestinal microorganisms of weaning piglets were investigated by adding IAMPs in diets, so as to provide reference for the development and utilization of IAMPs and make contributions to the cause of anti-resistance.

## Materials and methods

Immobilized antimicrobial peptide, purchased from CANGLUAN Biotech Co., Ltd., Zhengzhou, China was used.

### Experimental Design and Dietary Treatments

A total of 21 weaning piglets [Duroc ×(Landrace ×Yorkshire)] with initial body weight (7.64 ± 0.65 kg) were randomly allocated to one of three treatments with seven replicates (one pig per replicate) per treatment according to sex and weight in randomized complete block design. Pigs in the three treatments were fed corn–soybean meal-based diet (CON), corn–soybean meal-based diet + flavomycin (25 mg/kg) + quinone (50 mg/kg) (AB), and corn–soybean meal-based diet + 1,000 mg/kg IAMPs, respectively. The animal experiment lasted for 28 days, including early stage (0–14 days) and late stage (15–28 days). All the piglets had free access to the feed and water, while the living house of pigs was treated with pest control and immunization according to the routine procedures and was cleaned and disinfected regularly. The room temperature is automatically adjusted by a thermostatic controller, and the window is opened for ventilation at regular time. All the experimental diets meet the nutritional requirements of weaning piglets recommended by NRC (2012) (**Table 1**).

**Table 1:** Composition and nutrient levels of the experimental diet (%, as-fed basis).

Items	Content (%)
Ingredients	
Corn	54.75
Soybean meal	19.00
Full-fat soybean powder	10.00
Fish meal	5.00
Whey powder	6.15
Soybean oil	1.50
Dicalcium phosphate	0.90
L-Lysine–HCl, 78%	0.48
L-Threonine	0.05
DL-Methionine	0.10
L-Tryptophan	0.02
Salt	0.30
Limestone	0.50
Premix^a^	1.00
Cr_2_O_3_	0.25
Total	100.00
Calculated nutrients	
Digestible energy (MJ/kg)	14.64
Crude protein	20.15
Lysine	1.38
Methionine	0.82
Methionine + cysteine	1.01
Threonine	0.97
Tryptophan	0.25
Calcium	0.80
Total phosphorus	0.73

a The premix provided the following (per kilogram of complex feed): Vitamin A, 12,000 IU; Vitamin D, 2,500 IU; Vitamin E, 30 IU; Vitamin B12, 12 µg; Vitamin K, 3 mg; d-pantothenic acid, 15 mg; nicotinic acid, 40 mg; choline chloride, 400 mg; Mn, 40 mg; Zn, 100 mg; Fe, 90 mg; Cu, 8.8 mg; I, 0.35 mg; Se, 0.3 mg.

## Sample Collection and Chemical Analysis

### Growth Performance and Diarrhea Ratio

On days 0, 14, and 28 of the experiment, the body weight and feed intake of each piglet were recorded, and the feed was removed before 12 h of weighing. The average daily weight gain (ADG), the average daily feed intake (ADFI), and the ratio of gain:feed (G:F) of each piglet were calculated. The diarrhea occurrence of piglets was detected by visual assessment and was recorded by a trained researcher after careful daily observation. The fecal score assessment methods are as follows: 1 = normal feces; 2 = soft feces; 3 = partially formed soft feces; 4 = loose semi-liquid feces; and 5 = liquid feces separated by fecal water. Meanwhile, the diarrhea rate of piglets was calculated as follows: diarrhea rate (%) = total number of pigs with diarrhea/(number of pigs × feeding days) × 100.

### Serum Biochemical Index

Blood samples (about 5 ml) were collected from each pig *via* the jugular vein into 10-ml centrifuge tube on day 28 following an overnight fast. Samples were centrifuged (Biofuge 22R; Heraeus, Hanau, Germany) at 3,000×*g* for 15 min at 4°C, and the serum was kept at −20°C until analyzed. Serum total superoxide dismutase (T-SOD), glutathione peroxidase (GSH-Px), serum total antioxidative capacity (T-AOC), and malondialdehyde (MDA) content were determined with a commercial kit according to the supplier’s instructions (Nanjing Jiancheng Bioengineering Institute, Nanjing, China). Enzyme-linked immunosorbent assay (ELISA, Jiangsu Meimian Industrial Co. Ltd., Jiangsu, China) was used to measure the concentrations of serum IgA, IgG, IgE, and IgM.

### Serum Inflammatory Factors

Serum samples used in the above treatment process were used. The concentrations of serum anti-inflammatory factors including IL-4, IL-10, transforming growth factor (TGF-β), pro-inflammatory factor IL-12, tumor necrosis factor -α (TNF-α), and interferon γ (IFN-γ) were determined by using ELISA kit (Jiangsu Meimian Industrial Co. Ltd., China).

### Intestinal Barrier Function

D-Lactic acid, endotoxin concentrations, and diamine oxidase (DAO) activity in serum were determined using a pig ELISA kit (Jiangsu Meimian Industrial Co. Ltd., China). Specific test procedures were carried out according to the instructions provided by the supplier.

Total RNA of the jejunum mucosa was extracted by Trizol reagent extraction method, and cDNA was synthesized by reverse transcription according to the Evo M-MLV Mix Kit with gDNA Clean for quantitative PCR (qPCR) (Hunan Accurate Biological Co. Ltd., Changsha, China) procedure. β-Actin was used as the reference gene for real-time quantitative PCR, and the reaction condition was 95°C for 2 min, denaturation at 95°C for 10 s, annealing at 60°C, and extension for 40 s with 40 cycles. Fluorescence collection and melting curve were made according to the operation instructions of fluorescence quantitative PCR instrument (ABI PRISM 7500). The mRNA expression levels of occludin and ZO-1 were calculated by 2^−△△Ct^ method. The primer information is shown in **Table 2**, which was synthesized by Sangong Bioengineering Co., Ltd (Shanghai, China).

**Table 2:** Primer sequences information.

Target genes	Primer forward/reverse	Primer sequence (5’→3’)
β-actin	Forward	CTGCGGCATCCACGAAACT
	Reverse	AGGGCCGTGATCTCCTTCTG
ZO-1	Forward	CCTGAGTTTGATAGTGGCGTTGA
	Reverse	AAATAGATTTCCTGCTCAATTCC
Occludin	Forward	ACCCAGCAACGACATA
	Reverse	TCACGATAACGAGCATA

### Intestinal Morphology

At the end of the experiment, 21 weaning piglets were euthanized; the duodenum, jejunum, and ileum were separated and placed on a cooled stainless steel tray. The middle duodenum, proximal jejunum, and distal ileum were quickly cut out for about 5 cm, and then, the intestinal contents were gently washed with cold phosphate-buffered saline (PBS) solution. The clean intestine tissues were fixed in 10% formalin solution for intestinal morphology observation. The fixed intestine segments were dehydrated with ethanol (70%–100%); then, the sample was embedded in paraffin and made into 5-μm thick sections. Finally, xylene was used to clean and dewax for hematoxylin and eosin staining (HE staining). The morphological development of the small intestine was observed under a light microscope, and eight complete fields were selected from each section. Image J software was used to measure villus height and crypt depth of the small intestine and to calculate the ratios of villus height:crypt depth.

### Intestinal Microbiome Structure

According to the growth and health of weaning piglets, five pigs were randomly selected from each group, and the digesta of the cecum and colon were respectively collected for 16S rRNA high-throughput sequencing. Total DNA of digestas in the ileum, cecum, and colon was extracted by using Stool Mini Kit (Qiagen, Hilden, Germany); then, total DNA was detected with 1% agarose gel, and DNA concentration and purity were determined using a Nanodrop 2000 UV-VIS spectrophotometer (Thermo Fisher Scientific, Wilmington, USA). The specific primer with the barcode (16S V3–V4) with an ABI Gene Amp 9700 PCR thermocycler (ABI, CA, United States) was magnified, and the PCR products were extracted, purified, and quantified.

The amplified products were purified and sequenced by Illumina Miseq platform. Briefly, the raw 16S rRNA gene sequencing reads were demultiplexed, quality-filtered, and merged. Shannon and Chao indices were used as α-diversity parameters. Chao index can measure species richness, while Shannon index can measure species diversity (17). Principal coordinate analysis (PCoA) based on weighted_normalized_unifrac distance was used to evaluate the β-diversity. Meanwhile, analysis of similarities (ANOSIM) was adopted to evaluate the significant difference between samples.

RDP classifier was used to classify the flora of species, and operational taxonomic units (OTUs) representing <0.005% of the population were removed. Then, the relative abundance of each OTU was calculated at different levels of the biological group. Finally, bioinformatics analysis, OTU level calculation, β-diversity assessment, and comparison of differences between samples were performed in QIIME (V1.7.0) and Rpackages (V 3.2.0); meanwhile, the methods of the OTU table in QIIME, PCoA, and cluster analysis by ANOSIM were also used respectively to calculate and evaluate the β-diversity.

### Statistical Analysis

Statistical software (SAS 9.2) was used to conduct one-way analysis of variance (ANOVA) for data analysis. *p* ≤ 0.05 was considered as significant difference, *p ≤* 0.01 was considered as extremely significant difference, and 0.05 ≤ *p* < 0.10 was considered as trend.

## Results

### Effects of IAMPs on Growth Performance and Diarrhea Rate

**Table 3** shows that the final weight of piglets in the IAMPs group was significantly higher than that of the CON group (*p* < 0.05) and did not show any significant change when compared with the AB group. In the IAMPs group, the ADG at the later stage of the experiment (15–28 days) and during the whole experiment (0–28 days) was significantly increased (*p* < 0.05), but the ADG at the early stage of the experiment (0–14 days) did not significantly change compared with the AB group. The ADFI of weaning piglets was not affected by IAMPs supplementation. In the early (0–14 days), late (15–28 days), and whole period (0–28 days) of the experiment, the G:F ratios in the IAMPs group were significantly increased (*p* < 0.01) compared with the CON group, while there was no significant difference between the IAMPs and AB groups. The diarrhea rate in the early and whole period of the experiment of the IAMPs group was lower than that of the CON group, without showing significant difference when compared with the AB group, and the diarrhea rate in the late period of the experiment did not significantly change by the dietary treatments.

**Table 3:** Effects of IAMPs on growth performance and diarrhea rate of weaning piglets.

Items	Treatments			SEM	*p*-value
	CON	AB	IAMPs		
Day 0 BW (kg)	7.72	7.73	7.73	0.08	0.99
Day 14 BW (kg)	11.59	11.84	11.85	0.10	0.18
Day 28 BW (kg)	17.28^b^	18.35^a^	18.55^a^	0.32	0.03
Days 0-14					
ADG (g)	276.34	293.48	294.02	7.28	0.18
ADFI (g)	455.36	458.21	452.59	11.01	0.94
G:F	0.61^b^	0.64^a^	0.65^a^	0.01	<0.01
Diarrhea rate (%)	3.57^a^	0.89^b^	0.89^b^	0.34	<0.01
Days 15-28					
ADG (g)	406.43^b^	464.55^a^	478.48^a^	18.08	0.03
ADFI (g)	733.21	783.30	790.27	25.25	0.25
G:F	0.55^b^	0.59^a^	0.60^a^	0.01	<0.01
Diarrhea rate (%)	1.56	1.12	0.78	0.51	0.57
Days 0-28					
ADG (g)	341.38^b^	379.02^a^	386.25^a^	11.42	0.03
ADFI (g)	594.29	620.76	621.43	15.93	0.41
G:F	0.57^b^	0.61^a^	0.62^a^	0.01	<0.01
Diarrhea rate (%)	2.57^a^	1.00^b^	0.84^b^	0.37	0.01

SEM, standard error of the means (n = 7); ADG, average daily gain; ADFI, average daily feed intake; G:F, gain:feed; CON, corn–soybean based diet; AB, CON + 25 mg/kg flavomycin + 50 mg/kg quinocetone; IAMPs, CON + 1,000 mg/kg IAMPs.

^a,b^Different superscripts within a row indicate a significant difference (p < 0.05).

### Effects of IAMPs on Serum Biochemical Index of Weaning Piglets

As shown in **Table 4**, the IAMPs group had higher concentrations of serum IgE and IgG than the CON group (*p* < 0.05), without showing significant difference compared to the AB group. The concentration of serum IgM in IAMPs group was higher than that in the CON and AB groups (*p* < 0.05). The concentrations of serum IgA in the IAMPs group was not significantly changed compared to the other two treatments. Meanwhile, IAMPs diet significantly increased the concentration of serum GSH-Px compared to the CON group (*p* < 0.05) but not the AB group. The SOD concentration in the serum of IAMPs group was higher (*p* < 0.05) than that in the CON and AB groups, while the concentrations of MDA and T-AOC in serum were not significantly changed by dietary treatments.

**Table 4:** Effects of IAMPs on serum biochemical index of weaning pigs.

Items	Treatments			SEM	*p*-value
	CON	AB	IAMPs		
IgA, μg/ml	640.63	668.92	712.13	23.11	0.15
IgE, μg/ml	240.89^b^	267.64^a^	282.78^a^	7.32	0.01
IgG, μg/ml	18.10^b^	20.10^ab^	21.83^a^	0.79	0.03
IgM, μg/ml	17.05^c^	18.55^b^	23.17^a^	0.34	<0.01
SOD, ng/ml	176.60^b^	201.43^b^	231.17^a^	8.06	<0.01
GSH-Px, ng/ml	127.06^b^	143.89^a^	148.60^a^	4.92	0.03
MDA, nmol/ml	11.55	11.22	11.21	0.36	0.76
T-AOC, ng/ml	2.38	2.66	4.89	1.17	0.32

SEM, standard error of the means (n=7); IgA, immunoglobulin A; IgE, immunoglobulin E; IgG, immunoglobulin G; IgM, immunoglobulin M; SOD, superoxide dismutase; GSH-Px, glutathione peroxidase; MDA, malondialdehyde; T-AOC; total antioxidant capacity; CON, corn–soybean based diet; AB, CON + 25 mg/kg flavomycin + 50 mg/kg quinocetone; IAMPs, CON + 1000 mg/kg IAMPs.

^a,b,c^Different superscripts within a row indicate a significant difference (p < 0.05).

### Effects of IAMPs on Serum Inflammatory Factors of Weaning Piglets

**Table 5** shows that the concentrations of serum IL-10 and TGF-β during the whole experiment were higher in the IAMPs group than those in the CON group (*p* < 0.05) with no significant difference compared with the AB group. The serum IL-12 concentration was lower in the IAMPs group (*p* < 0.05) than that in the CON group, showing no significant difference compared with the AB group. The concentrations of serum TNF-α was lower in the IAMPs group (*p* < 0.05) than that in the CON and AB groups, while the concentrations of IL-4 and IFN-γ was not significantly affected by the dietary treatments.

**Table 5:** Effects of IAMPs on serum inflammatory factors of weaning piglets.

Items	Treatments			SEM	*p*-value
	CON	AB	IAMPs		
IL-10, pg/ml	252.01^b^	269.13^a^	285.05^a^	5.11	0.01
IL-12, pg/ml	126.97^a^	113.24^b^	115.01^b^	2.93	0.03
IL-4, pg/ml	42.33	44.96	47.08	1.85	0.25
IFN-γ, pg/ml	29.13	27.03	27.56	1.25	0.50
TNF-α, pg/ml	156.10^a^	150.70^a^	136.88^b^	4.06	0.03
TGF-β, pg/ml	675.22^b^	792.30^a^	757.48^a^	19.22	0.01

SEM, standard error of the means (n = 7); IL-10, interleukin 10; IL-12, interleukin 12; IL-4, interleukin 4; IFN-γ, γinterferon; TGF-β, transforming growth factor β; TNF-α, tumor necrosis factor α; CON, corn–soybean based diet; AB, CON + 25 mg/kg flavomycin + 50 mg/kg quinocetone; IAMPs, CON + 1,000 mg/kg IAMPs.

^a,b^Different superscripts within a row indicate a significant difference (p < 0.05).

### Effects of IAMPs on Intestinal Barrier Function of Weaning Piglets

As shown in **Table 6**, IAMPs diet induced lower serum endotoxin and D-lactic acid levels of weaning piglets compared with the CON diet (*p* < 0.05), while it did not significantly change serum DAO activity. There were no significant differences in DAO, endotoxin, and D-lactic acid levels between the AB and IAMPs groups.

**Table 6:** Effects of IAMPs on the concentrations of DAO, endotoxin, and diamine oxidase in serum of weaning piglets.

Items	Treatments			SEM	*p*-value
	CON	AB	IAMPs		
DAO, pg/ml	17.68	16.56	15.30	0.67	0.12
Endotoxins, EU/L	61.71^a^	52.43^b^	51.71^b^	2.29	0.04
D-Lactate, µmol/ml	107.71^a^	89.27^b^	90.93^b^	3.57	0.01

SEM, standard error of the means (n = 7); DAO, diamine oxidase; EU, endotoxin unit; CON, corn–soybean based diet; AB, CON + 25 mg/kg flavomycin + 50 mg/kg quinocetone; IAMPs, CON + 1,000 mg/kg IAMPs.

^a,b^Different superscripts within a row indicate a significant difference (p < 0.05).

**Figure 1:**
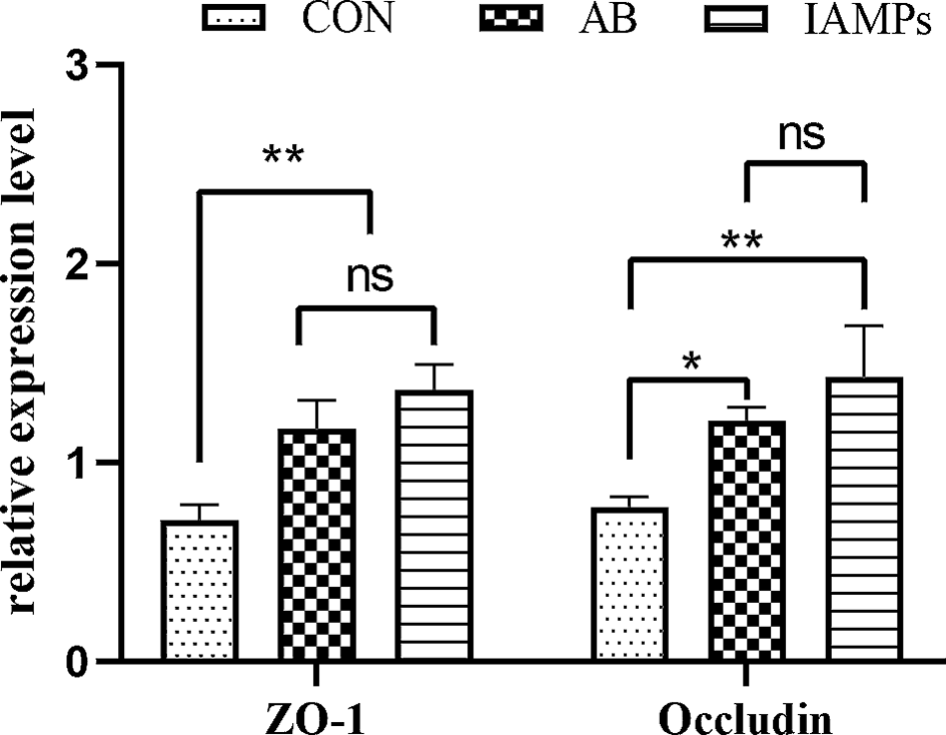
Effects of IAMPs on tight junction structure of jejunum (n = 7). CON, corn–soybean-based diet; AB, CON + 25 mg/kg flavomycin + 50 mg/kg quinocetone; IAMPs, CON + 1,000 mg/kg IAMPs. ns indicates *p* > 0.05; * indicates 0.05 < *p* < 0.01; ** indicates *p* < 0.01.

The relative expression levels of tight junction proteins including ZO-1 and Occludin in the jejunum mucosa of the piglets were determined by qPCR to verify the above experimental results. As shown in **Figure 1**, the relative expression of ZO-1 and occludin were significantly increased in the IAMPs group compared with that in the CON group (*p* < 0.05), but there was no significant difference in the expression of ZO-1 and occludin between the IAMPs and AB groups (*p* > 0.05).

### Effects of IAMPs on Intestinal Morphology of Weaning Piglets

As shown in **Table 7**, the villus height of the duodenum and jejunum was significantly increased in the IAMPs group compared with the CON group (*p* < 0.05) and without showing significant difference compared with the AB group. The dietary treatments did not significantly affect the villus height of the ileum. Moreover, IAMPs and AB diets did not show significant change in the crypt depth of the duodenum, jejunum, and ileum. The ratios of villus height:crypt depth of the duodenum and jejunum were significantly increased in the IAMPs group compared with that in the CON group (*p* < 0.05), while the ratios of villus height:crypt depth of the ileum was not significantly changed by the diets.

**Table 7:** Effects of IAMPs on intestinal morphology of weaning piglets.

Items	Treatments			SEM	*p-*value
	CON	AB	IAMPs		
Duodenum					
Villus height (µm)	357.43^b^	400.28^a^	408.09^a^	13.60	0.04
Crypt depth (µm)	265.01	262.25	260.09	2.72	0.46
Villus height/crypt depth	1.35^b^	1.53^a^	1.57^a^	0.05	0.03
Jejunum					
Villus height (µm)	316.85^b^	357.92^ab^	381.45^a^	13.82	0.02
Crypt depth (µm)	250.68	249.55	248.43	5.17	0.95
Villus height/crypt depth	1.26^b^	1.44^a^	1.53^a^	0.05	0.01
Ileum					
Villus height (µm)	321.36	342.03	348.40	8.39	0.10
Crypt depth (µm)	224.81	223.63	222.30	3.83	0.90
Villus height/crypt depth	1.43	1.53	1.57	0.05	0.13

SEM, standard error of the mean (n=7); CON, corn–soybean-based diet; AB, CON + 25 mg/kg flavomycin + 50 mg/kg quinocetone; IAMPs, CON + 1,000 mg/kg IAMPs.

^a,b^Different superscripts within a row indicate a significant difference (p < 0.05).

### Effects of IAMPs on Intestinal Microbiome Structure of Weaning Piglets

**Figure 2:**
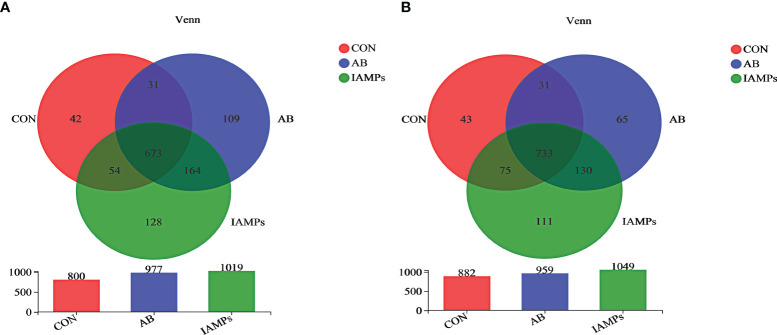
Effect of IAMPs on OTU for digesta samples of weaning piglets. **(A)** Cecum; **(B)** colon. The individual pig was regarded as the experimental unit (n = 5). CON, corn–soybean meal-based diet; AB, CON + 25 mg/kg flavomycin + 50 mg/kg quinocetone; IAMPs, CON + 1,000 mg/kg IAMPs.

**Figure 3:**
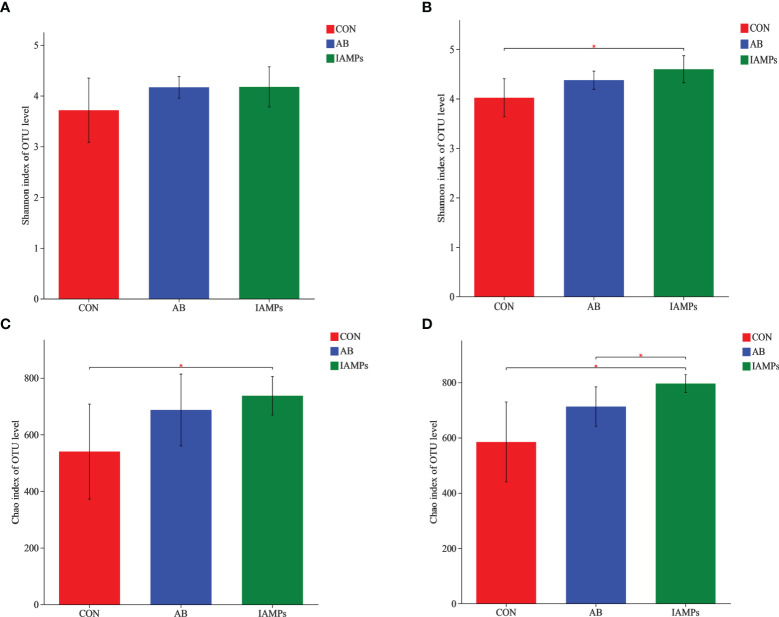
Effect of IAMPs on alpha-diversity index of cecal and colonic digesta of weaning piglets. **(A)** Cecal Shannon index; **(B)** cecal Chao index; **(C)** colonic Shannon index; **(D)** colonic chao index. The individual pig was regarded as the experimental unit (n = 5). CON, corn–soybean meal-based diet; AB, CON + 25 mg/kg flavomycin + 50 mg/kg quinocetone; IAMPs, CON + 1,000 mg/kg IAMPs. *indicates 0.05 < p < 0.01.

**Figure 4:**
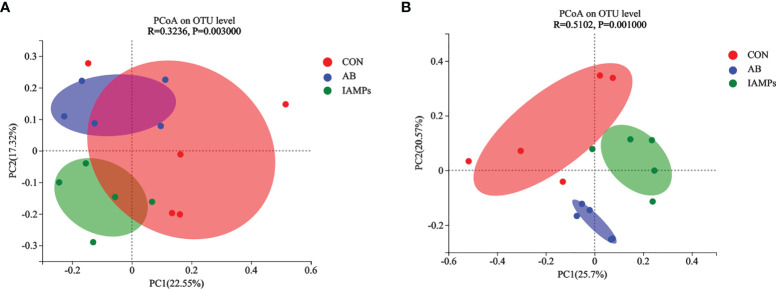
Effects of IAMPs on beta-diversity of cecal and colonic digesta of weaning piglets. **(A)** Cecum; **(B)** colon. The individual pig was regarded as the experimental unit (n = 5). CON, corn–soybean meal-based diet; AB, CON + 25 mg/kg flavomycin + 50 mg/kg quinocetone; IAMPs, CON + 1,000 mg/kg IAMPs.

Based on the results of 16S rRNA high-throughput sequencing, the OTU sequence similarity was 0.97, and the SilVA138/16s_Bacteria was used as taxonomic database with a taxonomic confidence of 0.97. The Rarefaction curves in **Supplementary Figure S1** indicated that each sample of digesta obtained from the cecum and colon had sufficient sequences for analysis. The OTU of cecal digesta in the three groups were 800, 977, and 1019, respectively, and the total OTU number was 673. Meanwhile, the OTU of colonic digesta in the CON, AB, and IAMPs groups were 882, 959, and 1049, respectively, of which 733 were common OTU. The number of OTU of both the cecal and colonic digesta in the AB and IAMPs groups tended to increase compared with that in the CON group (**Figure 2**). **Figure 3** shows the species richness and alpha diversity index of microbiota. Compared with the CON group, the Shannon index and the Chao index of cecal digesta were significantly increased in the IAMPs group (*p* < 0.05), and no significant difference was seen between the IAMPs and AB group. The Chao index of colonic digesta was significantly higher in IAMPs group than that in the CON and IAMPs groups (*p* < 0.05), and IAMPs had no significant effect on the Shannon index of cecal digesta. The dietary treatments resulted in significant changes in the beta diversity of the cecal and colonic microbiota as shown by PCoA of the weighted UniFrac distance metric (*p* < 0.01) (**Figure 4**).

**Figure 5:**
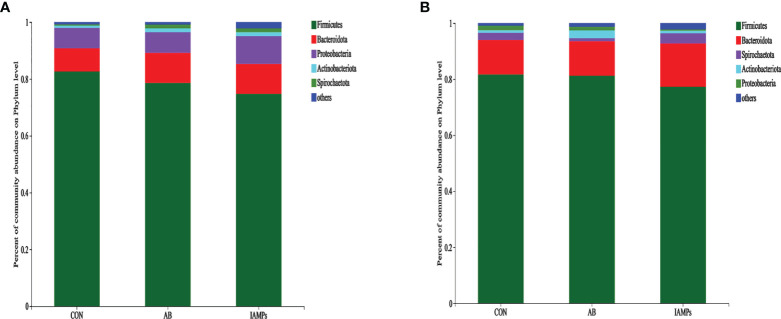
The composition of microbiota at the phylum level in the cecal and colonic digesta samples of weaning piglets. **(A)** Cecum; **(B)** colon. The individual pig was regarded as the experimental unit (n = 5). CON, corn–soybean meal-based diet; AB, CON + 25 mg/kg flavomycin + 50 mg/kg quinocetone; IAMPs, CON + 1,000 mg/kg IAMPs.

**Figure 6:**
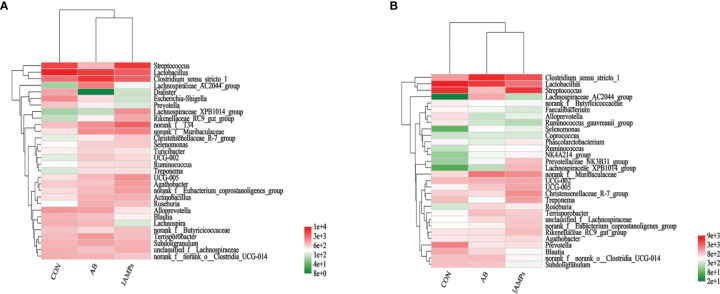
Heatmap of microbial community representing the top 30 bacteria at the genus level. **(A)** Cecum; **(B)** colon.The individual pig was regarded as the experimental unit (n = 5). CON, corn–soybean meal-based diet; AB, CON + 25 mg/kg flavomycin + 50 mg/kg quinocetone; IAMPs, CON + 1,000 mg/kg IAMPs.

At the phylum level, five dominant bacterial groups were identified in CON, AB, and IAMPs groups, including *Firmicutes*, *Proteobacteria*, *Bacteroidota*, *Actinobacteriota*, and *Spirochaetota* (**Figure 5**). The dominant flora of cecal digesta were *Firmicutes*, *Proteobacteria*, and *Bacteroidota*, accounting for 83.3%, 7.23%, and 8.26% in the CON group; 79.3%, 7.33%, and 10.7% in the AB group; and 76.4%, 9.98%, and 10.8% in the IAMPs group, respectively. *Firmicutes* and *Bacteroidota* were the two dominant bacterial communities in colonic digesta and accounted for 82.4% and 12.4% in the CON group, 82.3% and 12.5% in the AB group, and 79.0% and 15.8% in the IAMPs group, respectively. The richness of intestinal flora was reflected by color changes in the heatmap (**Figure 6**). At the genus level, the five major genera of the CON, AB, and IAMPs groups are *Streptococcus*, *Clostridium_sensu_stricto_1*, *Lactobacillus*, *Actinobacillus*, and *Escherichia–Shigella*. The relative abundance of *Escherichia–Shigella* in the cecal and colonic digesta was reduced in the IAMPs group compared with that in the CON group.

**Figure 7:**
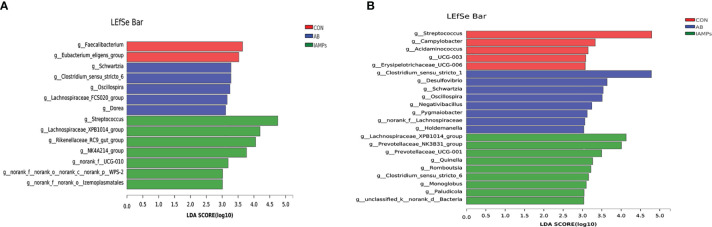
Identification of the most differentially abundant genera in the digesta samples of weaning piglets by linear discriminant analysis effect size (LEfSe) analysis. **(A)** cecum; **(B)** colon. The individual pig was regarded as the experimental unit (n = 5). CON, corn–soybean meal-based diet; AB, CON + 25 mg/kg flavomycin + 50 mg/kg quinocetone; IAMPs, CON + 1,000 mg/kg IAMPs.

LEfSe analysis was performed to assess the enrichment of taxa in the cecal and colonic digesta of the three dietary groups (**Figure 7**). In the cecal digesta, the relative abundance of *g_Clostridium_sensu_stricto*, *g_Schwartzia*, *g_Oscillospira*, *g_Lachnospiraceae_FCS020_group*, and *g_Dorea* were increased in the AB group, the relative abundance of *g_Faecalibacterium* and *g_Eubacterium_eligens_group* was increased in the CON group, and IAMPs group showed increased relative abundance of *g_Streptococcus*, *g_Lachnospiraceae_XPB1014_group*, *G_Rikenellaceae_RC9_gut_group*, *g_NK4A214_group*, *g_NORank_F_UCG-010*, *g_norank_f_norank_o_Izemoplasmatales*, *g_Norank_F_NORank_O*, and *g_NORank_C_norank_P_WPS-2*. In the colonic digesta, the CON group had increased relative abundance of *g_Streptococcus*, *g_Campylobacter*, *g_Acidaminococcus*, and *G_UCG-003*, the AB group showed increased relative abundance of *g_Clostridium_sensu_stricto_1*, *g_Desulfovibrio*, *g_Schwartzia*, *g_Oscillospira*, *g_Negativibacillus*, and *g_Holdemanella*, and the IAMPs group had increased relative abundance of *Lachnospiraceae_XPB1014_group*, *g_Prevotellaceae_NK3B31_group*, *g:prevotellaceae_UCG-001*, *g_Romboutsia g_Clostridium_sensu_STRICto_6*, *g_Paludicola*, *g_Monoglobus*, and *g_Acetitomaculum*.

## Discussion

Weaning piglets have undeveloped gastrointestinal tract and experience “weaning stress” due to the sudden change in diet and living environment; as a result, diarrhea usually occurs, which negatively affects the growth performance and even leads to death (2). In this experiment, diet supplemented with IAMPs increased the ADG and G:F ratios of weaning piglets and reduced the diarrhea rate of weaning piglets. This was also observed by previous studies, which found that adding compound AMPs in the diet improved the growth performance of piglets, thus increasing the effective utilization rate of feed and bringing more economic benefits to farmers (18, 19). In addition, adding antimicrobial peptide WK3 in the diet attenuated diarrhea symptoms of ETEC K88 challenged pig models and improved growth performance of piglets (20). The results of this experiment are consistent with the results of the studies mentioned above, in which adding IAMPs in the diets of weaning piglets improved the growth performance and attenuated the diarrhea rate of piglets.

In this experiment, IAMPs supplementation significantly improved the concentrations of IgG, IgE, and IgM in serum of piglets; meanwhile, it increased the concentrations of antioxidant indexes such as GSH-Px and SOD in serum. Consistent with the results of the current study, Yuan et al. (21) reported that diet supplemented with AMPs improved IgA, IgG, and IgM levels in serum of weaning piglets, thus enhancing humoral immune response and reducing the occurrence of diseases in weaning piglets. Studies have shown that the AMPs can activate the immune function of the body. For instance, Tang et al. (22) found that dietary supplementation of fusion peptide bovine lactoferricin–lactoferrampin improved the activities of GSH-Px and T-AOC in serum and liver of piglets and increased the concentrations of IgA, IgE, and IgM in serum of piglets, which indicated an improved immune capacity of piglets. Ren et al. (23) showed that AMPs reduced the level of MDA and improved T-AOC, GSH-Px, and SOD activities in the spleen of piglets. Serum immunoglobulin level can reflect the immune status and liver health status of the body, while T-SOD is associated with the integrity and barrier function of the cell membrane, and GSH-Px, T-AOC, and MDA are closely related to antioxidant capacity (24, 25). Therefore, the improved serum immunoglobin levels and antioxidant indices by dietary IAMPs supplementation indicated enhanced humoral immunity and antioxidant capacity of weaning piglets, which was beneficial for weaning piglets to resist pathogens, reduce the occurrence of diseases, and ensure the health status, and could be a good option to substitute antibiotics.

The intestinal tract of weaned piglets is not fully developed and is easily infected by pathogens; therefore, an improved immune response can contribute to maintain the intestinal health (26, 27). Cytokines including inflammatory factors, growth factors, and chemokines play a role in the proliferation and differentiation of immune and non-immune cells and also play a significant role in the regulation of intestinal inflammation (28). Most pro-inflammatory cytokines, such as IL-6, IFN-γ, and TNF-α, can increase intestinal epithelial permeability, while anti-inflammatory cytokines, such as IL-10 and TGF-β can maintain intestinal barrier integrity (29, 30). In this study, IAMPs significantly increased the concentrations of IL-10 and TNF-β and decreased the concentrations of IL-12 and TNF-α in serum. Generally, the levels of pro-inflammatory cytokines such as IL-6 and TNF-α in the intestinal tract of weaning piglets after weaning are upregulated, which destroys the integrity of the intestinal epithelium and causes intestinal inflammation (31, 32). Similar with our findings, Yi et al. (33) found that cathelicidin-BF reduced the concentrations of anti-inflammatory cytokines including IL-6, IL-22, and TNF-α in serum of piglets by intraperitoneal injection of Cathelicidin-BF into weaning piglets at 21 days of age. Moreover, tracheal antimicrobial peptide was also found to react against pathogens and produce antibacterial substances to regulate inflammatory factors such as TNF-α and IL-1β (34). Meanwhile, compared with enrofloxacin, Cathelicidin-WA reduced diarrhea rates and alleviated intestinal inflammation by showing downregulated IL-6, IL-8, and IL-22 levels in weaning piglets with diarrhea (35, 36). The changes in inflammatory factors in serum indicated that IAMPs reduced the proinflammatory response and enhanced the anti-inflammatory response of weaning piglets, and the improved immune capacity could be linked with the improved growth performance and diarrhea rates of piglets.

Intestinal barrier function plays a fundamental role in intestinal health and diseases. Increased intestinal permeability can lead to the occurrence of intestinal diseases such as inflammatory and functional bowel diseases (37–39). In this experiment, IAMPs significantly reduced the levels of serum endotoxin and D-lactic acid and increased the relative expressions of ZO-1 and occludin in the jejunum. Changes in DAO activity, endotoxin, and D-Lactate concentrations in serum and the relative expression of ZO-1 and occludin are important indicators reflecting intestinal permeability and intestinal barrier injury and repair (40–42). When the intestinal barrier is damaged, the increased permeability leads to increased DAO levels secreted by intestinal epithelial cells and increased bacterial metabolites D-lactate and endotoxin concentrations produced by intestinal flora (43). Xiao et al. (18, 44) found that adding compound AMPs in the diet of weaning piglets counteracted the effects of deoxynivalenol on intestinal permeability and reduced serum D-lactate and DAO concentrations. Yu et al. (11) reported that the addition of antimicrobial peptide Microcin J25 significantly reduced the levels of D-lactate, DAO, and endotoxin in serum of weaning piglets, showing a similar effects compared with an antibiotic group. Tang et al. (45) found that oral administration of antimicrobial peptide Buforin II in piglets infected with *E. coli* increased the relative expression of occludin and ZO-1 and decreased the concentrations of D-lactate and DAO. Song et al. (46) found experimentally that Cathelicidin-BF could eliminate the effect of LPS on intestinal barrier and increase the expression levels of ZO-1 and occludin in the jejunum. In agreement with this study, these studies illustrated that IAMPs could maintain the integrity of the intestinal barrier, which could be a contributor for the improved growth performance and reduced occurrence of diarrhea in piglets.

Villus height and crypt depth are important indicators of intestinal morphology. In this study, IAMPs improved the villus height of the duodenum and jejunum, and the villus height:crypt depth ratio were significantly increased, which indicated increased mature intestinal cells and the stronger ability to digest and absorb nutrients (47–49). This was also observed by Cao et al. (50) in that adding antimicrobial peptide WK3 in diet of weaning piglets can significantly increase ileal villus height and villus height:crypt depth ratio, and the ratio is even higher than that in the antibiotic group. Studies also reported that AMPs showed similar effects on intestinal morphology compared to antibiotics; they significantly improved villus height and the villus height:crypt depth ratio in the duodenum and jejunum (51–53). These conclusions are consistent with this study and suggested that IAMPs maintained the morphology of the small intestine, which might improve nutrient digestion and absorption capacity, thus promoting the performance of piglets.

Gut microbes are involved in a variety of physiological responses, and the composition of gut microbiota plays an important role in gut health and immune system development (54, 55). In this study, it was found that IAMPs significantly increased the Chao index in cecal and colonic digesta and the Shannon index in the colon, indicating that the diversity and richness of intestinal flora were increased. Previously, supplementation of *Lactobacillus plantarum PFM 105* was reported to increase the Shannon index of microbiota in the colon of weaning piglets, which was explained to be related to an enhanced disease resistance of weaning piglets (56). Besides the improved species diversity of microbiota, the relative abundance of beneficial bacteria such as *Bacteroidota* and *Lachnospiraceae* was increased in the colon and cecum, while some harmful bacteria such as *Escherichia–Shigella*, *Campylobacter*, and *Faecalibacterium* decreased in the colon and cecum of IAMPs group. After weaning, the homeostasis between beneficial and pathogenic microorganisms in the intestine of piglets is broken, which reduces immunity, results in diarrhea and intestinal inflammation, and consequently affects feed intake and decreases growth performance of piglets (57). *Lachnospiraceae* ferments carbohydrates to produce short-chain fatty acids that protect intestinal cells from injury (58). *Bacteroidota* can decompose polysaccharides to improve nutrient utilization, closely related to systematic immunity, and plays a pivotal role in maintaining intestinal microecological balance (59, 60). *Escherichia–Shigella* secretes toxins after colonizing in the intestine and causes metabolic disorders of intestinal epithelial cells, thereby damaging intestinal mucosal structure and resulting in intestinal immune dysfunction that induced acute diarrhea (61). Tang etal. (22) found that the relative abundance of *Escherichia–Shigella* in the ileum, cecum, and colon decreased by adding AMPs to the diets of weaning piglets. Meanwhile, a previous study illustrated that antimicrobial peptide-P5 reduced the relative abundance of *Escherichia–Shigella* in the cecum of weaning piglets (62). This observation confirmed our hypothesis that the IAMPs could relieve intestinal flora disorder caused by the weaning stress, maintain intestinal health, and reduce the occurrence of intestinal diseases in weaning piglets.

## Conclusion

In conclusion, dietary supplementation of 1,000 mg/kg IAMPs can improve ADG and G:F of weaning piglets, reduce diarrhea rate, increase serum immunoglobulin concentrations, increase species diversity and richness of intestinal flora, and decrease the relative abundance of *Escherichia–Shigella*, thereby effectively improving the growth performance, antioxidant and immune capacity, and intestinal health of piglets. Overall, IAMPs can be used as an option to replace antibiotics to attenuate weaning stress of weaning piglets.

## Data Availability Statement

The datasets presented in this study can be found in online repositories. The names of the repository/repositories and accession number(s) can be found below: https://www.ncbi.nlm.nih.gov/sra/PRJNA800167.

## Ethics Statement

The animal study was reviewed and approved by the Animal Care Committee of Hunan Agricultural University (Changsha, China).

## Author Contributions

NL: conceptualization, methodology, and software. XM: literature collection and writing original draft preparation. XM and XJ: writing, reviewing, and editing. XM: funding acquisition. All authors contributed to the article and approved the submitted version.

## Funding

This research was supported by Hunan Provincial Natural Science Foundation of China (2021JJ30318), the "Open Project Program of Key Laboratory of Feed Biotechnology, the Ministry of Agriculture and Rural Affairs of the People's Republic of China", and the China Agriculture Research System of MOF and MARA, Earmarked Fund for China Agriculture Research System (CARS-35).

## Conflict of Interest

The authors declare that the research was conducted in the absence of any commercial or financial relationships that could be construed as a potential conflict of interest.

## Publisher’s Note

All claims expressed in this article are solely those of the authors and do not necessarily represent those of their affiliated organizations, or those of the publisher, the editors and the reviewers. Any product that may be evaluated in this article, or claim that may be made by its manufacturer, is not guaranteed or endorsed by the publisher.
