# Supplementary material for: Effects of Immobilized Antimicrobial Peptides on Growth Performance, Serum Biochemical Index, Inflammatory Factors, Intestinal Morphology, and Microbial Community in Weaning Pigs
Source: Front Immunol. 2022 Mar 29;13:872990. doi: 10.3389/fimmu.2022.872990 (PMC9001916; doi:10.3389/fimmu.2022.872990)
Supplement: Supplementary file 1 [file Table_1.docx]

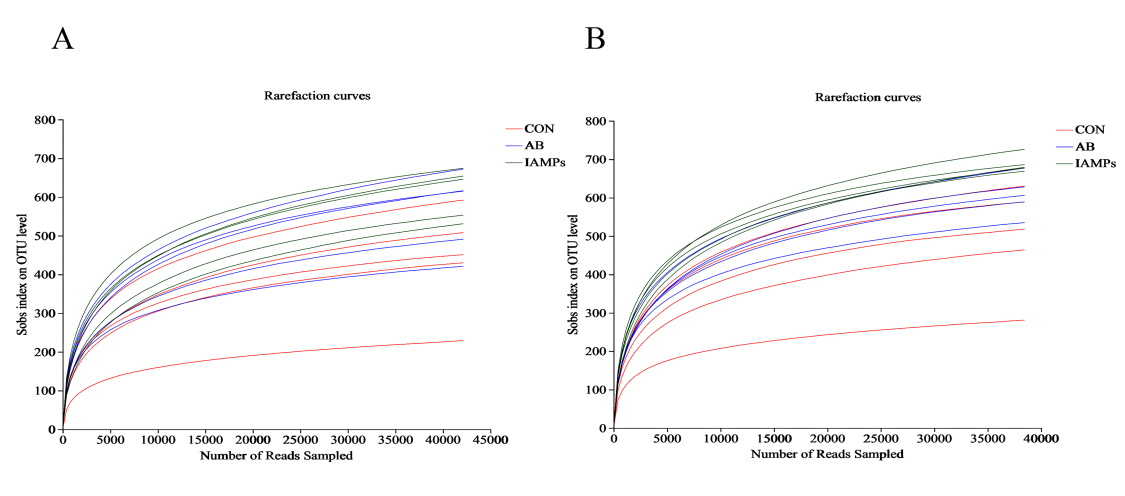


**Supplementary Figure 1.** Rarefaction curve for digesta samples of weaning piglets fed with CON, AGP and AB diets. A: cecum; B:colon . The individual pig was regarded as the experimental unit (n = 5). CON: corn-soybean meal based diet; AB: CON + 25 mg/kg flavomycin + 50 mg/kg quinocetone; IAMPs: CON + 1000 mg/kg IAMPs.
